# School absenteeism among school‐aged children with medically attended acute viral respiratory illness during three influenza seasons, 2012‐2013 through 2014‐2015

**DOI:** 10.1111/irv.12440

**Published:** 2017-02-15

**Authors:** Huong Q. McLean, Siri H. Peterson, Jennifer P. King, Jennifer K. Meece, Edward A. Belongia

**Affiliations:** ^1^ Marshfield Clinic Research FoundationMarshfieldWIUSA

**Keywords:** absenteeism, children, influenza

## Abstract

**Background:**

Acute respiratory illnesses (ARIs) are common in school‐aged children, but few studies have assessed school absenteeism due to specific respiratory viruses.

**Objective:**

To evaluate school absenteeism among children with medically attended ARI due to common viruses.

**Methods:**

We analyzed follow‐up surveys from children seeking care for acute respiratory illness who were enrolled in the influenza vaccine effectiveness study at Marshfield Clinic during the 2012‐2013 through 2014‐2015 influenza seasons. Archived influenza‐negative respiratory swabs were retested using multiplex RT‐PCR to detect 16 respiratory virus targets. Negative binomial and logistic regression models were used to examine the association between school absence and type of respiratory viruses; endpoints included mean days absent from school and prolonged (>2 days) absence. We examined the association between influenza vaccination and school absence among children with RT‐PCR‐confirmed influenza.

**Results:**

Among 1027 children, 2295 days of school were missed due to medically attended ARIs; influenza accounted for 39% of illness episodes and 47% of days missed. Mean days absent were highest for influenza (0.96‐1.19) and lowest for coronavirus (0.62). Children with B/Yamagata infection were more likely to report prolonged absence than children with A/H1N1 or A/H3N2 infection [OR (95% CI): 2.1 (1.0, 4.5) and 1.7 (1.0, 2.9), respectively]. Among children with influenza, vaccination status was not associated with prolonged absence.

**Conclusions:**

School absenteeism due to medically attended ARIs varies by viral infection. Influenza B infections accounted for the greatest burden of absenteeism.

## 1 Introduction

Acute respiratory illnesses (ARIs) are common in school‐aged children, with approximately 30‐40% affected during the winter months in the United States.^1^ Influenza accounts for the majority of the ARIs in this age group,^2^, ^3^ but other viruses such as respiratory syncytial virus (RSV), human metapneumovirus, and parainfluenza virus also circulate in the winter. School‐aged children with influenza tend to miss more school than those with respiratory illnesses of other etiologies.^4^, ^5^ Few studies have assessed the burden of school absenteeism due to laboratory‐confirmed influenza,^5^, ^6^, ^7^ and only one was conducted after the recommendation for annual influenza vaccination in children in the United States.^5^ Absenteeism data on the relative contribution of ARIs caused by viruses other than influenza are lacking. Non‐influenza viral illnesses may be less common, but also likely to disrupt usual activities and cause increased school absenteeism. Quantifying school absenteeism due to specific viruses can help target prevention or treatment strategies to reduce burden in school‐aged children.

We utilized data from an observational influenza vaccine effectiveness study to evaluate parental‐reported school absenteeism across three seasons among children with medically attended ARI due to various viruses. Specifically, we aim to estimate the average days absent for specific respiratory viruses in children, identify risk factors for prolonged (>2 days) absence from school due to viral ARIs, and evaluate the association between influenza vaccination and prolonged absence among children with influenza.

## 2 Methods

We conducted an analysis using follow‐up surveys from children with medically attended ARIs who were enrolled in the US Influenza Vaccine Effectiveness Network Study (influenza VE study) at Marshfield Clinic during the 2012‐2013, 2013‐2014, and 2014‐2015 influenza seasons.^8^, ^9^, ^10^ Prior to each influenza season, a community cohort was defined, consisting of approximately 50,000 residents of Marshfield, Wisconsin, and surrounding rural areas. The racial and ethnic demographic for this region is predominately White (97%) with approximately 4% of Hispanic ethnicity. Throughout the influenza season, research coordinators recruited patients during outpatient visits for ARIs. Individuals in the defined cohort were eligible for recruitment to the influenza VE study if they were ≥6 months old and presented with symptoms of cough lasting no more than seven days at the time of their visit. Consenting patients completed an enrollment interview and provided a nose and throat swab for influenza testing. From the enrollment interviews, we obtained information on age, race/ethnicity, self‐reported health status prior to the onset of illness, illness onset date, and symptoms. Vaccination status was obtained from the validated vaccine registry that serves the population.^11^

Approximately 1 week following enrollment, all influenza‐positive patients and approximately 50 influenza‐negative patients per week were contacted for a follow‐up interview. For children, interviews were conducted with a parent or guardian and included questions about when the child returned to normal activities, how many days the child missed school due to the illness (numerically open‐ended), and medications prescribed and taken. The answer to the question, “how many days of school did your child miss, due to this illness” was used to assess school absenteeism. Responses larger than the maximum number of weekdays between illness onset and follow‐up interviews were truncated to the maximum weekdays within the interval (nine observations were truncated).

### 2.1 Laboratory testing

Combined nose and throat swabs collected at the time of enrollment in the influenza VE study were tested for influenza virus (type and subtype) using real‐time reverse‐transcription polymerase chain reaction (RT‐PCR).^12^ All patients with swabs testing positive for influenza were notified of their results within 2 days of their clinic visit. After influenza testing, samples were archived and frozen. For this study, all available archived samples negative for influenza were retested using a multiplex respiratory virus panel (eSensor^®^ Respiratory Viral Panel; GenMark Diagnostics, Inc., Carlsbad, CA, USA), as previously described.^13^ The panel tested for RSV A and B, human rhinovirus, human metapneumovirus, parainfluenza viruses 1‐4, coronaviruses OC43, NL63, HKU1, and 229E, adenoviruses B, C, and E, and influenza A and B. Due to resource constraints, influenza‐positive samples were not retested for coinfections in two out of the three seasons. As a result, we were unable to identify all coinfections with influenza. Samples initially testing negative for influenza that tested positive for influenza and another viral infection with the multiplex respiratory virus panel were classified under the appropriate influenza category. Participants negative for influenza, but positive for >1 other viruses tested, were classified as “coinfection.” Participants negative for all viruses tested were classified as “no virus detected.”

Study procedures were approved by the Institutional Review Board (IRB) at the Marshfield Clinic Research Foundation. Informed consent/assent was obtained from all participants at the time of enrollment into the influenza VE study. The additional multiplex testing was subsequently approved by the IRB with a waiver of informed consent.

### 2.2 Statistical analysis

For this study, we included children 5‐17 years old at the time of enrollment in the influenza VE study, whose parent or guardian had participated in the follow‐up interview and who attended school outside the home.

Demographic and clinical characteristics were compared using χ^2^ tests. Absenteeism rate was estimated by dividing the total number of days missed by the number of children with the infection. Negative binomial regression models were used to estimate the average days absent and 95% confidence interval (CI) for each viral infection category because absenteeism was skewed to the right. All variables potentially associated with days absent were added to the initial model. These variables included sex, race/ethnicity, reported health status prior to current illness, influenza vaccination status at the time of illness onset, and receipt of antivirals (after enrollment in the influenza VE study). Backward elimination with a cutoff of *P* value=.05 was conducted to determine inclusion in the final model. Age was included a priori. Children with coinfections with viruses other than influenza were excluded.

Similar multivariate methods (with the same variables listed above) were performed to identify risk factors for prolonged absence from school using logistic regression. Age was included a priori. Prolonged absence was defined as >2 days absent from school.

We performed a separate analysis to estimate the mean days absent for vaccinated and unvaccinated children and examine the association of influenza vaccination and prolonged absence due to influenza. This analysis was restricted to children infected with influenza, and each influenza subtype and lineage (A/H1N1, A/H3N2, B/Yamagata, and B/Victoria) were evaluated separately. Children who were vaccinated within 14 days of illness onset or were not considered adequately vaccinated according to the Advisory Committee on Immunization Practices (ACIP) were excluded.^14^, ^15^, ^16^ We conducted analyses (i) removing data from the 2014–2015 season for A/H3N2, since antigenically drifted A/H3N2 3C.2a viruses predominated in the study population and the vaccine was not effective against the drifted virus,^10^ and (ii) removing children who received live attenuated influenza vaccine (LAIV) for A/H1N1, since the A/H1N1pdm09 component of the LAIV vaccine was not effective during the 2013‐2014 season.^17^, ^18^

All analyses were performed using sas statistical software (version 9.3; SAS Institute, Cary, NC, USA).

## 3 Results

During the 2012‐2013 through 2014‐2015 influenza seasons, 1082 (76%) of 1423 children 5‐17 years old enrolled in the influenza VE study at Marshfield Clinic had a completed follow‐up interview. Respondents and non‐respondents were similar with regard to age, sex, race/ethnicity, reported symptoms, influenza vaccination status, and time from illness onset to influenza VE study enrollment (data not shown). Respondents were more likely to be enrolled in the influenza VE study during the period after influenza circulation peaked (63% vs 46%, *P*<.0001). Among respondents, 55 (5%) were excluded because they did not attend school outside the home (n=37), had influenza B with no lineage data (n=12), had influenza A and B or B/Yamagata and B/Victoria coinfection and could not be classified into a single influenza virus group (n=3), or had missing data on days absent from school (n=3).

**Figure 1:**
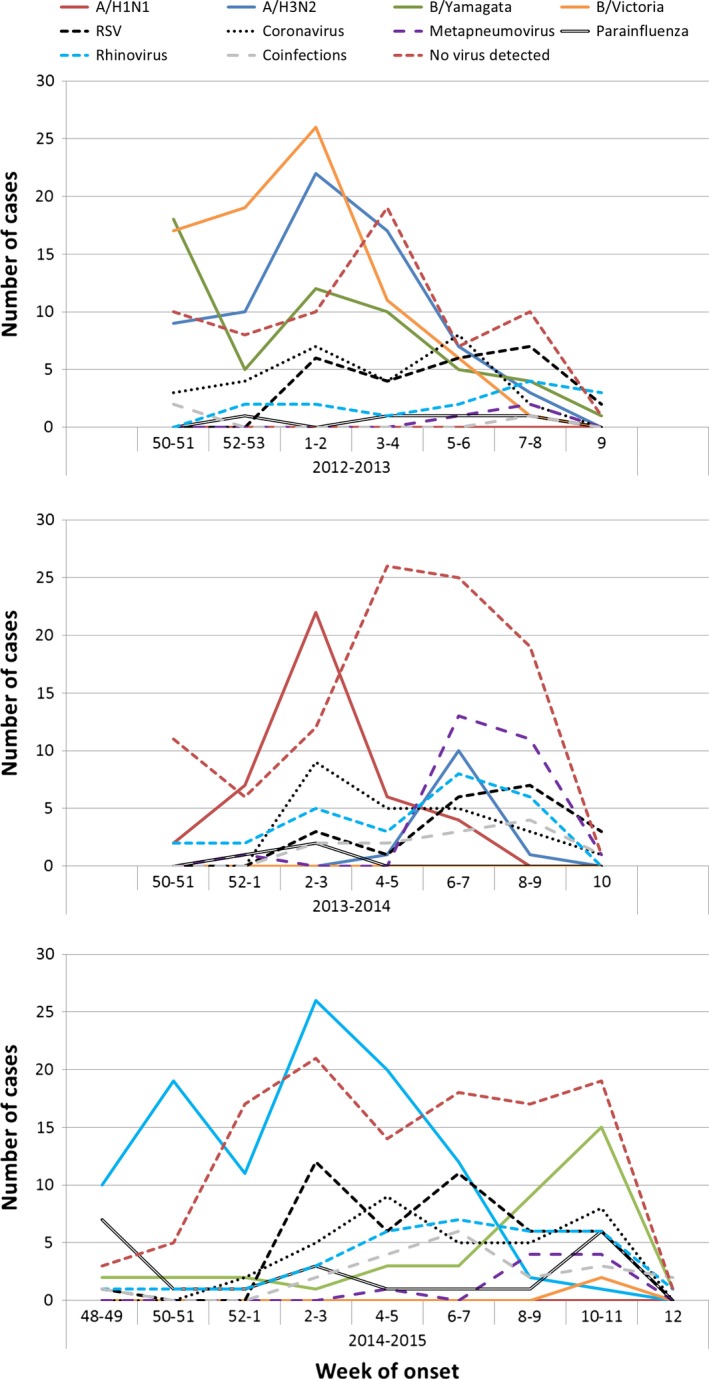
Distribution of cases by week of onset and influenza season

Among the 1027 children included in this study, viral infections were detected in 747 (73%) (Table 1). Overall, 181 (18%) had influenza A/H3N2, 93 (9%) had influenza B/Yamagata, 85 (8%) had coronavirus (OC43=39, NL63=32, 229E=10, HKU1=6), 82 (8%) had influenza B/Victoria, 77 (7%) had RSV (RSV B=51, RSV A=26), 72 (7%) had rhinovirus, 41 (4%) had influenza A/H1N1, 38 (4%) had metapneumovirus, 28 (3%) had parainfluenza virus (parainfluenza 2=13, parainfluenza 3=10, parainfluenza 1=3, parainfluenza 4 = 2), three (0.3%) had adenovirus (adenovirus C=3), 45 (4%) had viral coinfections that were not influenza, and 282 (27%) had no viral infections detected. Figure 1 shows the distribution of cases by week of onset. Influenza A/H1N1 was only present during the 2013‐2014 season, while influenza A/H3N2, B/Yamagata, and B/Victoria occurred during 2012‐2013 and 2014‐2015. Single detection of RSV, coronavirus, parainfluenza virus, and adenovirus was more likely during 2014‐2015. Metapneumovirus infections were more likely during 2013‐2014.

**Table 1:** Demographic and Clinical Characteristics by Viral Infection^a^

Characteristic	A/H1N1 (n=41)	A/H3N2 (n=181)	B/Yamagata (n=93)	B/Victoria (n=82)	RSV (n=77)	Coronavirus^b^ (n=85)	Metapneumovirus (n=38)	Parainfluenza (n=28)	Rhinovirus (n=72)	Coinfections^c^ (n=45)	No viral infection detected (n=282)
Season											
2012‐2013	0	68 (38)	55 (59)	80 (98)	19 (25)	28 (33)	3 (8)	4 (14)	14 (19)	9 (20)	67 (24)
2013‐2014	41 (100)	12 (7)	0	0	16 (21)	23 (27)	26 (68)	3 (11)	26 (36)	16 (36)	100 (35)
2014‐2015	0	101 (56)	38 (41)	2 (2)	42 (55)	34 (40)	9 (24)	21 (75)	32 (44)	20 (44)	115 (41)
Age, years											
5‐8	20 (49)	71 (39)	38 (41)	47 (57)	40 (52)	27 (32)	25 (66)	12 (43)	32 (44)	34 (76)	82 (29)
9‐17	21 (51)	110 (61)	55 (59)	35 (43)	37 (48)	58 (68)	13 (34)	16 (57)	40 (56)	11 (24)	200 (71)
Sex (male)	23 (56)	89 (49)	53 (57)	45 (55)	39 (51)	40 (47)	19 (50)	11 (39)	37 (51)	31 (69)	154 (55)
Race/ethnicity^d^											
White	36 (88)	168 (92)	84 (91)	69 (84)	66 (86)	79 (93)	34 (89)	26 (93)	66 (92)	40 (89)	248 (88)
Hispanic	2 (5)	4 (2)	5 (5)	6 (7)	5 (6)	3 (4)	1 (3)	1 (4)	3 (4)	1 (2)	18 (6)
Other	3 (7)	9 (5)	3 (3)	7 (9)	6 (8)	3 (4)	3 (8)	1 (4)	3 (4)	4 (9)	15 (5)
Reported general health status											
Excellent	26 (63)	110 (61)	50 (54)	47 (57)	53 (69)	44 (52)	23 (61)	14 (50)	34 (47)	26 (58)	155 (55)
Very good/good	14 (34)	68 (38)	42 (45)	34 (41)	23 (30)	41 (48)	15 (39)	14 (50)	35 (49)	19 (42)	122 (43)
Fair/poor	1 (2)	3 (2)	1 (1)	1 (1)	1 (1)	0	0	0	3 (4)	0	5 (2)
Vaccinated^e^	15 (37)	81 (45)	12 (13)	32 (39)	39 (51)	38 (45)	24 (63)	11 (39)	29 (40)	19 (42)	127 (45)
Received IIV^f^	5 (33)	59 (74)	10 (83)	27 (84)	27 (69)	22 (61)	16 (67)	10 (91)	23 (79)	13 (68)	81 (65)
Received LAIV^f^	10 (67)	21 (26)	2 (17)	5 (16)	12 (31)	14 (39)	8 (33)	1 (9)	6 (21)	6 (32)	44 (35)
Time of season^g^											
Pre‐peak influenza weeks	3 (7)	13 (7)	18 (19)	17 (21)	0	3 (4)	0	2 (7)	2 (3)	2 (4)	22 (8)
Peak influenza weeks	28 (68)	65 (36)	26 (28)	51 (62)	11 (14)	23 (27)	1 (3)	10 (36)	13 (18)	4 (9)	63 (22)
Post‐peak influenza weeks	10 (24)	103 (57)	46 (53)	14 (17)	66 (86)	59 (69)	37 (97)	16 (57)	57 (79)	39 (87)	197 (70)
Symptoms											
Fatigue	37 (90)	174 (96)	86 (92)	78 (95)	66 (86)	71 (83)	35 (92)	25 (89)	60 (83)	35 (78)	233 (83)
Fever	38 (93)	161 (89)	85 (91)	78 (95)	50 (65)	45 (53)	22 (58)	17 (61)	35 (49)	25 (56)	161 (57)
Nasal congestion	35 (85)	154 (85)	73 (78)	75 (91)	66 (86)	73 (86)	29 (76)	20 (71)	64 (89)	41 (91)	208 (74)
Shortness of breath	11 (27)	70 (39)	42 (45)	32 (39)	26 (34)	32 (38)	11 (29)	18 (64)	23 (32)	11 (24)	90 (32)
Sore throat	34 (83)	130 (72)	79 (85)	55 (67)	54 (70)	66 (78)	24 (63)	25 (89)	59 (82)	32 (71)	224 (79)
Wheezing	14 (34)	56 (31)	26 (28)	28 (34)	22 (29)	22 (26)	13 (34)	9 (32)	23 (32)	14 (30)	53 (19)
Median duration of illness, days (IQR)	6 (5, 9)	6 (4.5, 9)	7 (6, 9)	7 (6, 9)	7 (5, 9)	5 (4, 8)	6 (5, 8)	5 (1, 9)	6 (4, 8.5)	6 (5, 8)	6 (4, 8)
Interval from illness onset to follow‐up											
7‐10 d	31 (76)	112 (62)	57 (61)	48 (59)	40 (52)	46 (54)	22 (58)	18 (64)	43 (60)	21 (46)	156 (55)
11‐15 d	10 (24)	69 (38)	36 (39)	34 (41)	37 (48)	39 (46)	16 (42)	10 (36)	29 (40)	24 (53)	126 (45)
Received antivirals^h^	4 (10)	29 (16)	5 (5)	11 (13)	1 (1)	5 (6)	0	0	2 (3)	0	14 (5)

Data are no. (%) of total.

a Three adenovirus infections not included.

b Includes two coinfections with the same virus: cnl63 and coc43, c229e and coc43.

c Coinfections include RSV and coronavirus (7), rhinovirus and coronavirus (7), coronavirus and metapneumovirus (6), adenovirus and rhinovirus (4), RSV and metapneumovirus (4), RSV and adenovirus (3), RSV and rhinovirus (3), RSV and parainfluenza (3), adenovirus and coronavirus (2), metapneumovirus and rhinovirus (2), parainfluenza and rhinovirus (1), parainfluenza and coronavirus (1), RSV, rhinovirus, and coronavirus (1), adenovirus, metapneumovirus, and rhinovirus (1).

d Missing race/ethnicity information for two participants, one positive for influenza B/Yamagata and one with no infection detected; % reported among those with race/ethnicity information.

e 17 were considered partially vaccinated according to ACIP recommendations, and nine were vaccinated within 14 d of illness onset.

f % reported among vaccinated with vaccine‐type information; five missing data on vaccine type.

g Relative to influenza season.

h Missing antiviral information for two participants, one positive for influenza A/H3N2 and one with no infection detected; % reported among those with race/ethnicity information.

Abbreviations: RSV, respiratory syncytial virus; IQR, interquartile range.

The age distribution varied across viral categories. Coinfections and infections with metapneumovirus were more common in children 5‐8 years old relative to older children, while having no viral infection detected and coronavirus was more common in children 9‐17 years old. There were no differences in sex, race/ethnicity, or reported health status prior to infection between viral groups. Less than half (42%) of all subjects had received influenza vaccine prior to illness onset. Fever was more commonly reported among children with influenza infection than those with other viral infection or no viral infection detected. There was no difference in duration of illness or time from illness onset to completion of follow‐up survey across viral categories; the majority (58%) of subjects completed their follow‐up interviews within 7‐10 days of illness onset.

### 3.1 Mean days absent

In total, 2295 days of school were missed by our study population over three influenza seasons; 175 (17%) children did not miss any days. The 2012‐2013 season, reported fair/poor health status prior to illness, enrollment during the peak influenza season, and receipt of antivirals were associated with greater mean days absent from school (Table 2). Longer duration of illness, being fatigued, having a fever, being short of breath, and having a sore throat were also associated with greater mean days absent. There were no differences in mean days absent by age group, sex, race/ethnicity, or influenza vaccination status.

**Table 2:** Demographic and clinical characteristics by days absent from school

	N	Mean days absent (95% CI)	*P* a	Prolonged absence^b^ N (%)	*P* c
Season					
2012‐2013	338	0.90 (0.82, 0.98)	.001	161 (48)	<.0001
2013‐2014	247	0.65 (0.55, 0.76)		74 (30)	
2014‐2015	394	0.79 (0.71, 0.87)		141 (36)	
Age					
5‐8 y	394	0.78 (0.70, 0.86)	.5	153 (39)	.8
9‐17 y	585	0.81 (0.75, 0.88)		223 (38)	
Sex					
Female	510	0.79 (0.72, 0.86)	.9	193 (38)	.7
Male	469	0.80 (0.73, 0.87)		183 (39)	
Race/ethnicity					
White	876	0.78 (0.73, 0.84)	.2	332 (38)	.5
Hispanic	52	0.97 (0.76, 1.18)		19 (39)	
Other	49	0.86 (0.64, 1.08)		24 (46)	
Reported general health status					
Excellent	556	0.75 (0.68, 0.81)	.05	201 (36)	.2
Very good/good	408	0.85 (0.78, 0.93)		168 (41)	
Fair/poor	15	1.04 (0.67, 1.41)		7 (47)	
Vaccination Status					
Unvaccinated	571	0.82 (0.76, 0.89)	.2	221 (39)	.8
Vaccinated	408	0.76 (0.68, 0.84)		155 (38)	
Received IIV	280	0.78 (0.68, 0.87)	.6	106 (38)	.9
Received LAIV	123	0.73 (0.58, 0.87)		47 (38)	
Time of season^d^					
Pre‐peak influenza weeks	80	0.30 (0.10, 0.51)	<.0001	20 (25)	.005
Peak influenza weeks	291	0.88 (0.79, 0.97)		129 (44)	
Post‐peak influenza weeks	608	0.81 (0.75, 0.87)		227 (37)	
Symptoms					
Fatigue					
Yes	865	0.85 (0.79, 0.90)	<.0001	355 (41)	<0.0001
No	114	0.34 (0.16, 0.51)		21 (18)	
Fever					
Yes	692	0.91 (0.86, 0.97)	<.0001	314 (45)	<.0001
No	287	0.45 (0.35, 0.55)		62 (22)	
Nasal congestion					
Yes	797	0.81 (0.76, 0.87)	.3	315 (40)	.1
No	182	0.74 (0.62, 0.86)		61 (34)	
Shortness of breath					
Yes	355	0.87 (0.79, 0.95)	.03	149 (42)	.08
No	624	0.76 (0.69, 0.82)		227 (36)	
Sore throat					
Yes	750	0.83 (0.77, 0.88)	.05	297 (40)	.2
No	229	0.70 (0.60, 0.81)		79 (34)	
Wheezing					
Yes	266	0.88 (0.78, 0.97)	.06	115 (43)	.06
No	713	0.77 (0.71, 0.83)		261 (37)	
Duration of illness					
<4 d	540	0.69 (0.62, 0.76)	<.0001	166 (31)	<.0001
5‐7 d	228	0.82 (0.72, 0.93)		100 (44)	
≥8 d	211	1.01 (0.92, 1.11)		110 (52)	
Interval from illness onset to follow‐up					
7‐10 d	573	0.81 (0.74, 0.88)	.6	223 (39)	.7
11‐15 d	406	0.78 (0.70, 0.86)		153 (38)	
Antiviral use					
Received antivirals	71	0.99 (0.82, 1.17)	.03	34 (48)	.09
No antivirals	906	0.78 (0.73, 0.83)		342 (38)	

a *P*‐value based on the limiting chi‐square distribution from the negative binomial regression model.

b Prolonged absence defined as >2 d absent.

c *P*‐value from chi‐square test.

d Relative to influenza season.

**Figure 2:**
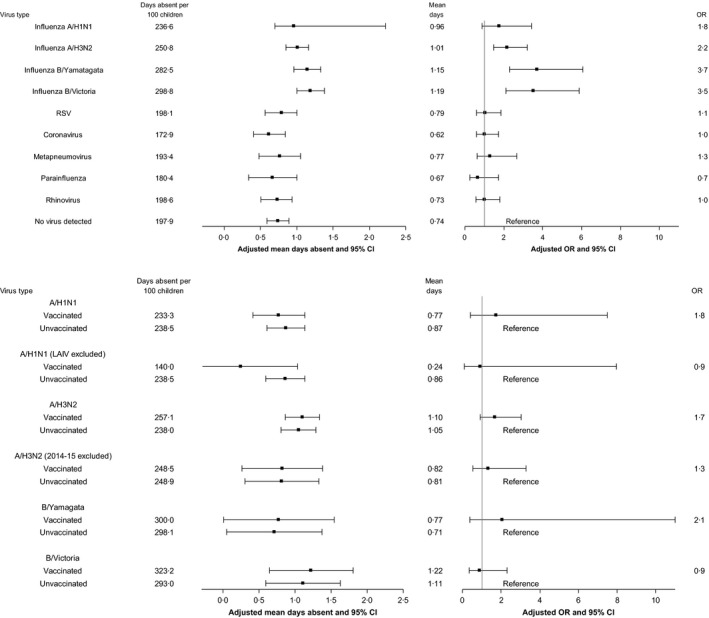
Adjusted^a^ mean days absent and prolonged absence by viral infection. 

^a^Adjusted for age and health status in negative binomial regression models and age in logistic regression models. Panel A excludes participants with adenovirus and coinfections. Panel B excludes children who were vaccinated within 14 d of illness onset or were not considered adequately vaccinated according to the Advisory Committee on Immunization Practices

Influenza infection accounted for 39% of ARI visits and 47% of all days absent (A/H3N2, 20%; B/Yamagata, 12%; B/Victoria, 11%; A/H1N1, 4%). Those with no infection detected accounted for 24%, and other viruses accounted for between <1% (adenovirus) and 7% (RSV) of all days absent. Mean days absent was highest for influenza and was not significantly different by subtype or lineage (Figure 2A, range: 0.96‐1.19). Mean days absent was higher among children with influenza A/H3N2, B/Yamagata, and B/Victoria compared to those with RSV (*P*≤.04), coronavirus (*P*≤.0004), parainfluenza (*P*≤.04), and rhinovirus (*P*≤.01). Among children with influenza, there was no difference in mean days absent between vaccinated and unvaccinated children with medically attended influenza (Figure 2B). When we excluded children vaccinated with LAIV, the mean days absent due to A/H1N1 among vaccinated children was lower, but confidence intervals overlapped the mean days absent for all vaccinated children. For H3N2, exclusion of the 2014‐2015 season resulted in lower mean days absent for both vaccinated and unvaccinated children.

### 3.2 Risk factors for prolonged absence

Most children (62%) missed two or fewer days of school due to their medically attended ARI. The 2012‐2013 season and peak influenza season were associated with prolonged absence of >2 days (Table 2). Longer duration of illness, being fatigued, and having a fever were also associated with prolonged absence. There were no differences in prolonged absence by age group, sex, reported health status prior to illness, influenza vaccination status, or receipt of antivirals.

More than 40% of children with influenza reported prolonged absence (B/Yamagata 60%, B/Victoria 59%, A/H3N2 47%, A/H1N1 41%) compared to other viruses (range: from 21% for parainfluenza to 34% for human metapneumovirus). The odds of experiencing prolonged absence was 3.7 (95% CI: 2.3, 6.1), 3.5 (95% CI: 2.1, 5.9), and 2.2 (95% CI: 1.5, 3.2) for influenza B/Yamagata, B/Victoria, and A/H3N2, respectively, compared to children with no viral infection (Figure 2A). Children with B/Yamagata infection were more likely to report prolonged absence than children with A/H1N1 (OR=2.1, 95% CI: 1.0, 4.5, *P*=.05) or A/H3N2 (OR=1.7, 95% CI: 1.0, 2.9, *P*=.04) infection. Vaccination status was not associated with prolonged absence among children with influenza (Figure 2B). When we excluded children vaccinated with LAIV, the odds of prolonged absence due to A/H1N1 among vaccinated children was attenuated, but confidence intervals overlapped the odds for all vaccinated. Results for A/H3N2 did not change when the 2014‐2015 season was excluded.

Results for influenza did not change in sensitivity analysis excluding the 20 known coinfections with influenza.

## 4 Discussion

In this study, we evaluated the association between specific viral infections and school absence. Influenza, RSV, coronaviruses, and rhinoviruses were the most commonly detected viruses in children 5‐17 years old with medically attended acute respiratory illness, and viral illness accounted for about 75% of missed school days. Influenza contributed to a substantial burden on school absenteeism, averaging over one missed school day per illness and over 40% missing >2 days. School absenteeism due to ARI caused by other viruses contributed to fewer days missed, but was common.

Influenza accounted for almost half of days absent by children with medically attended acute respiratory illness. Seasons predominated by A/H3N2 tend to be associated with increased morbidity and mortality,^19^, ^20^, ^21^ but there was no difference in the average days missed due to influenza between children with different subtypes or lineage in our study population. However, children with influenza B/Yamagata infection were more likely to have prolonged absence than children with influenza A. The reason for this is unclear; influenza B infection rates are highest in school‐aged children, and those with influenza B were more likely to seek medical attention,^1^, ^22^ but more cases of A/H3N2 were identified in our study. In contrast, previous studies have found lower, but similar absenteeism rates by influenza type^6^, ^7^ and are consistent with studies indicting similar clinical features among children with influenza A and B.^7^, ^23^, ^24^, ^25^

Influenza vaccination did not impact school absenteeism among children with influenza in our study. Previous studies have found substantially lower absenteeism rates among vaccinated children compared to unvaccinated children, but these studies examined absenteeism due to non‐specific respiratory illness rather than laboratory‐confirmed influenza, and residual confounding may have occurred.^4^, ^26^ School absenteeism rates were also lower in schools/communities with school‐based vaccination programs.^5^, ^27^, ^28^, ^29^, ^30^ These studies did not assess the reason for absenteeism or were limited to influenza‐like illness. It is possible that vaccination may have resulted in milder illness, leading to fewer outpatient visits. Our study population was restricted to respiratory illnesses that were severe enough to seek medical attention, and was not able to assess mild illness due to vaccination that may have resulted in fewer school days missed. However, a prior randomized placebo‐controlled trial in children with influenza B found no difference in duration of illness between those receiving the vaccine and those receiving a placebo.^31^

For children under five years old, RSV causes significant morbidity, with hospitalization rates higher than those reported for influenza.^32^, ^33^, ^34^ For older children and adults, RSV typically causes more mild illness than influenza.^13^ In our study, the prevalence of RSV infection and school absenteeism due to RSV was lower than that among children with influenza, consistent with milder illness compared to influenza. However, this contrasts a previous study among children attending the emergency room in Italy, where there was no difference in median days missed from school between children infected with RSV and influenza.^35^ The median days missed were much higher (10‐12 days) than those in our outpatient study, suggesting emergency room visits may have been more severe in general and may explain discrepancies between the two studies.

RSV, coronavirus, and rhinovirus were prevalent during all seasons examined, but these viruses contributed to fewer missed school days. This is not surprising as coronaviruses and rhinoviruses are the most frequently identified viruses, but are less likely to be medically attended.^1^ Although the prevalence of metapneumovirus was low, estimated mean days missed due to metapneumovirus was similar to RSV, consistent with a study that found similar school absenteeism rates among children with metapneumovirus and children with RSV.^35^ While most children are infected with these viruses before the age of 5 years, reinfection is common and contributes to school absenteeism among school‐aged children.

This study had several limitations. First, patients testing positive for influenza were notified of test results within 2 days of study enrollment, prior to their follow‐up interview. Parent's knowledge of their child's influenza status may have affected their behavior and school attendance. Knowledge of a known illness may have kept the child out of school longer than an illness without confirmation of etiology. Second, our study likely underestimated the burden of school absenteeism due to respiratory illness because our study population was restricted to children with medically attended illness and did not assess children who did not seek care. In a household cohort, 38% of ARIs occurred in school‐aged children and approximately 20% of all ARI cases were medically attended.^1^ The proportion medically attended varied by viral etiology. Additionally, enrollment for our study was restricted to the influenza season and did not capture viral respiratory illnesses that occurred outside this period, likely underestimating the burden of medically attended illnesses due to viruses other than influenza. However, including only medically attended illnesses would lead to overestimation of the average duration of absenteeism. Finally, we did not test for coinfections among those identified with influenza from the influenza VE study in all seasons or bacterial pathogens.

During the influenza season, excess illness and school absenteeism is expected.^36^, ^37^ Our study confirmed the substantial burden of influenza on school‐aged children, but likely underestimates school absenteeism due to other and less‐severe viral respiratory illnesses. We noted increased school absenteeism among children with medically attended influenza compared to children with other viral infections, including RSV, but no differences in school absenteeism among children with medically attended influenza by vaccination status. Additional studies are needed among children who develop influenza despite vaccination to determine whether influenza vaccine can reduce disease severity and duration, and subsequently school absenteeism. RSV vaccines are currently undergoing pre‐licensure clinical trials in both children and adults. Data on school absenteeism due to RSV should be considered when estimating the direct and indirect impact of potential RSV vaccine policies.
